# Developmental Changes Within the Genetic Architecture of Social Communication Behavior: A Multivariate Study of Genetic Variance in Unrelated Individuals

**DOI:** 10.1016/j.biopsych.2017.09.020

**Published:** 2018-04-01

**Authors:** Beate St Pourcain, Lindon J. Eaves, Susan M. Ring, Simon E. Fisher, Sarah Medland, David M. Evans, George Davey Smith

**Affiliations:** a Language and Genetics Department, Max Planck Institute for Psycholinguistics, The Netherlands; b Donders Institute for Brain, Cognition and Behaviour, Radboud University, Nijmegen, The Netherlands; c Medical Research Council Integrative Epidemiology Unit, University of Bristol, Bristol, United Kingdom; d School of Social and Community Medicine, University of Bristol, Bristol, United Kingdom; e Department of Human and Molecular Genetics, Institute for Psychiatric and Behavioral Genetics, Commonwealth University School of Medicine, Richmond, Virginia; f Psychiatric Genetics, QIMR Berghofer Medical Research Institute, Queensland, Australia; g University of Queensland Diamantina Institute, Translational Research Institute, University of Queensland, Brisbane, Queensland, Australia

**Keywords:** ALSPAC, Genetic relationship matrix, Genetic-relationship matrix structural equation modeling, Genetic variance decomposition, Longitudinal analysis, Structural equation modeling

## Abstract

**Background:**

Recent analyses of trait-disorder overlap suggest that psychiatric dimensions may relate to distinct sets of genes that exert maximum influence during different periods of development. This includes analyses of social communication difficulties that share, depending on their developmental stage, stronger genetic links with either autism spectrum disorder or schizophrenia. We developed a multivariate analysis framework in unrelated individuals to model directly the developmental profile of genetic influences contributing to complex traits, such as social communication difficulties, during an approximately 10-year period spanning childhood and adolescence.

**Methods:**

Longitudinally assessed quantitative social communication problems (*N* ≤ 5551) were studied in participants from a United Kingdom birth cohort (Avon Longitudinal Study of Parents and Children; age range, 8–17 years). Using standardized measures, genetic architectures were investigated with novel multivariate genetic-relationship-matrix structural equation models incorporating whole-genome genotyping information. Analogous to twin research, genetic-relationship-matrix structural equation models included Cholesky decomposition, common pathway, and independent pathway models.

**Results:**

A two-factor Cholesky decomposition model described the data best. One genetic factor was common to Social Communication Disorder Checklist measures across development; the other accounted for independent variation at 11 years and later, consistent with distinct developmental profiles in trait-disorder overlap. Importantly, genetic factors operating at 8 years explained only approximately 50% of genetic variation at 17 years.

**Conclusions:**

Using latent factor models, we identified developmental changes in the genetic architecture of social communication difficulties that enhance the understanding of autism spectrum disorder– and schizophrenia-related dimensions. More generally, genetic-relationship-matrix structural equation models present a framework for modeling shared genetic etiologies between phenotypes and can provide prior information with respect to patterns and continuity of trait-disorder overlap.

SEE COMMENTARY ON PAGE 544

The extent to which genetic etiologies are shared between traits and disorders naturally depends on the genetic composition of the two phenotypes. While psychiatric disorders are diagnostic entities defined by clinical criteria, including the age of onset, human behavior changes continously during development. This includes developmental alterations in complex genetic trait architectures as reported for cognitive (1) as well as social communication–related characteristics (2).

Difficulties in socially engaging and communicating with others, as observed in the general population, are heritable (twin-h^2^ = 0.74) (3), and a considerable proportion of the underlying genetic variation can be tagged by single nucleotide polymorphism (SNP) heritability (SNP-h^2^ ≤ 0.45) (2). For both social communication and social interaction problems, multivariate twin 4, 5 and bivariate genetic-relationship-matrix residual maximum likelihood (GREML) studies (6) reported evidence for a degree of genetic stability but also change during childhood and adolescence 2, 7, 8 that may affect genetic similarities with other traits.

Studying the genetic overlap between psychatric illness and social communication difficulties across multiple developmental stages, different developmental profiles for childhood-onset versus adult-onset psychiatric disorders have been identified (9). The genetic overlap with clinical autism spectrum disorder, a complex, highly heritable early-onset neurodevelopmental condition (10), was strongest for social communication difficulties during childhood but declined with progressing age of the trait. By contrast, the genetic correlation with clinical schizophrenia, an adult-onset psychiatric illness with a typical first-time diagnosis between 16 and 30 years of age (10), was highest for social communication problems during later adolescence (9). Thus, the risk of developing these contrasting psychiatric conditions might be related to distinct sets of genes, both of which affect social communication skills but exert their maximum influence during different periods of development.

Discontinuity in trait-disorder overlap may, however, also result because of attrition-related artifacts, such as decreasing power or inherent sample bias (11). As knowledge about developmental changes in complex genetic trait architectures is still scarce, development-related variations in trait-disorder overlap are often dismissed. The aim of this study is to provide insight into the developmental profile of genetic factors influencing complex traits, such as social communication difficulties during childhood and adolescence, using a longitudinal analysis framework. Building on our previous work 2, 9, we investigate here two extreme hypotheses, as follows: 1) whether the genetic variance/covariance structure of social communication difficulties during childhood and adolescence is consistent with multiple independent genetic influences, suggesting developmental changes in the genes responsible for interindividual variation over time, or 2) whether, alternatively, there is evidence for a shared single genetic factor, irrespective of age.

To study the developmental profile of genetic factors in unrelated individuals, we implemented multivariate genetic-relationship-matrix structural equation models (GSEMs). These models use genome-wide genetic relationship matrices (GRMs) (12), calculated from hundreds of thousands of SNPs across the genome, to estimate the total amount of phenotypic variance and covariance tagged by common genetic variants, similar to GREML 12, 13. GREML and related approaches 12, 14, 15, 16 have reshaped the research of complex genetic trait architectures beyond twin designs by exploiting the availability of genome-wide genetic data in cohorts of unrelated individuals. Genetic correlations are, however, typically estimated by these methods by studying two phenotypes only. Using a structural equation modeling (SEM) framework (17), as widely applied within twin research 4, 5, we now extend this bivariate approach by flexibly modeling complex latent genetic factor structures within a multivariate context. In this article, we use multivariate GSEMs to model longitudinal data on social communication difficulties across childhood and adolescence in the Avon Longitudinal Study of Parents and Children (ALSPAC), a phenotypically rich longitudinal population-based birth cohort from the United Kingdom (18).

## Methods and Materials

### Participants and Measures

All analyses were carried out using children’s data from ALSPAC, a United Kingdom population-based longitudinal pregnancy-ascertained birth cohort (estimated birth dates 1991–1992) (18). The study website contains details of all the data that are available through a fully searchable data dictionary (http://www.bris.ac.uk/alspac/researchers/data-access/data-dictionary/). Ethical approval was obtained from the ALSPAC Law and Ethics Committee (IRB00003312) and the local research ethics committees. Written informed consent was obtained from a parent or individual with parental responsibility, and assent (and for older children consent) was obtained from the child participants.

#### Phenotype Information

Social communication difficulties during childhood and adolescence were collected with the 12-item mother-reported Social Communication Disorder Checklist (SCDC) (score range, 0–24; age range, 3–18 years) (3). The SCDC is a brief screening instrument of social reciprocity and verbal and nonverbal communication (e.g., “Not aware of other people’s feelings”), which has high reliability and internal consistency and good validity (3), with higher scores reflecting more social communication deficits. Quantitative SCDC scores in ALSPAC children and adolescents were measured at 8, 11, 14, and 17 years of age, and information on phenotypic and genotypic data was available for 4174 to 5551 children (Supplemental Table S1).

Descriptive analyses of SCDC scores were performed with R version 3.2.4 (R Foundation for Statistical Computing, Vienna, Austria). The distribution of SCDC scores was positively skewed and predominantly leptokurtic (Supplemental Table S1). Each score was adjusted for sex, age, and the two most significant ancestry-informative principal components (see below) using ordinary least squares regression. Residuals were subsequently transformed to perfect normality using rank-based inverse normal transformation (19), as previously reported (9), to allow for comparisons across different algorithms (see below). There were moderate phenotypic correlations between repeatedly assessed SCDC scores using both untransformed and transformed data (SCDC, Spearman’s rho, 0.39–0.57; Pearson’s *r*, 0.38–0.61) (Supplemental Table S2) as previously shown (9).

#### Genome-wide Genotype Information

ALSPAC children were genotyped using the Illumina HumanHap550 quad chip genotyping platforms (Supplemental Methods). After quality control, 8237 children and 477,482 directly genotyped SNPs were kept within the study.

### Genetic-Relationship-Matrix Structural Equation Models

Multivariate SEM techniques were used to assess the relative importance of genetic and residual influences to variation in longitudinal SCDC scores during child and adolescent development. Similar to GREML (12), GSEMs use the genetic similarity between unrelated individuals to partition the expected phenotypic variance/covariance matrix into genetic and residual components. More generally, however, the statistical framework of GSEM is analogous to twin analysis methodologies 4, 5 but uses GRMs, instead of twin correlations, to estimate genetic variance/covariance structures using full information maximum likelihood (FIML). Thus, genetic and environmental influences are modeled in the GSEM framework as latent factors contributing to interindividual covariation in phenotypic measures. The advantage of our approach is that multivariate SEM methodology has been widely established within twin research 4, 5 and allows for flexible modeling of complex genetic factor structures. Conversely, GREML, as implemented in the Genome-wide Complex Trait Analysis (GCTA) software package, is currently restricted to bivariate situations (6). While multivariate GSEMs can be fit with SEM software such as OpenMx (20) using both mxGREML and FIML algorithms, these models are currently computationally expensive (see Results). We therefore implemented GSEMs within R (version 3.2.4) (for details see Supplemental Methods).

In short, GSEMs describe the phenotypic covariance structure using one or more additive genetic factors A that capture genetic variance, tagged by common genotyped SNPs, as well as one or more residual factors E that capture residual variance, containing untagged genetic variation and unique environmental influences (including measurement error). As SEM methodology has its origins in the method of path analysis (21), path diagrams are useful in visualizing the relationship among observed and latent variables (represented as squares and circles, respectively). In these diagrams, single-headed arrows (factor loadings or paths) denote causal relationships between measures, whereas double-headed arrows define correlations.

In our formulation, additive genetic variances (GSEM-Var_g_) and genetic covariances are modeled as the product of additive genetic factor loadings and genetic factor variances (the latter being standardized to unit variance). For example, using multivariate GSEM, a saturated model can be fit to the data through a decomposition of both the genetic variance and the residual variance into as many latent factors as there are observed variables (Cholesky decomposition model) (Supplemental Methods). Estimated genetic variances and covariances can then be used to estimate genetic correlations (GSEM-r_g_) (22), i.e., the extent to which two phenotypes share common genetic factors (Supplemental Methods). We used the Cholesky decomposition model as a saturated and baseline model (Supplement). Besides Cholesky decomposition models, multivariate GSEMs also permit the fitting of models with smaller numbers of latent genetic and residual factors, defined according to theory (23).

Multivariate GSEMs of longitudinally assessed SCDC scores were fitted in two stages. In the first stage (I), we specified a priori three standard multivariate AE models, analogous to twin research: a Cholesky decomposition model (saturated model), an independent pathway model, and a common pathway model.

1. The Cholesky decomposition model, as described above, is a fully parameterized descriptive model without any restrictions on the structure of latent genetic and residual influences (20 free parameters) and involves multiple independent genetic influences sharing genetic etiologies across development.
2. The independent pathway model, in its simplest form, specifies a single common genetic factor and a single common residual factor, in addition to age-specific genetic and residual influences (16 free parameters).
3. The common pathway model, in its simplest form, parameterizes a single latent factor, influenced by both genetic and residual sources of variance, in addition to age-specific genetic and residual influences, and is the most constrained model (14 free parameters). The model constrains the variance of the latent factor to one (i.e., the sum of squared genetic and residual factor loadings). Although the likelihood of this model can be estimated, the resulting Hessian is not invertible owing to singularity problems. For these reasons, the model constraint was relaxed within this work.

Both the independent pathway model and the common pathway model are consistent with a shared single genetic factor across development and are nested submodels of the full Cholesky decomposition model. The goodness of fit of GSEMs to empirical data was assessed using likelihood ratio test (LRT), the Akaike information criterion (24), and the Bayesian information criterion (25) (Supplemental Methods).

In the second stage (II), we adopted a data-driven approach and investigated the pattern of genetic factor loadings for the best-fitting model from stage I in detail. The smallest genetic factor loadings were successively dropped from the model, and the overall fit of the model was compared with the best-fitting a priori defined GSEM (or an adapted form) using LRTs. The statistical significance of factor loadings was assessed using a Wald test (two-sided test). Standard errors for genetic and residual variances and covariances and genetic correlations were derived from the variance-covariance matrix of the estimated factor loadings using the delta method. Standard errors for factor loadings were estimated by GSEMs. For rank-transformed measures with unit variance, such as the SCDC scores in this study, genetic variances are equivalent to SNP-h^2^ estimates. However, path coefficients for multivariate GSEMs were restandardized to enhance the interpretability.

GRMs were estimated using the GCTA software (12) and based on directly genotyped SNPs. All GSEMs were fitted to data from participants with nonmissing information to simplify the estimation algorithm. All R scripts are available via the R gsem package (https://gitlab.gwdg.de/beate.stpourcain/gsem) (Supplement). For the purpose of benchmark comparisons with univariate GCTA, we also fitted univariate GSEMs, where genetic variances were estimated as a single variance component.

### Genetic-Relationship-Matrix Residual Maximum Likelihood

The GCTA software package can be used to estimate the proportion of phenotypic variation that is jointly explained by SNPs on a genotyping chip using GREML (AE model) (13). Likewise, bivariate GREML (6) allows estimating genetic covariances and genetic correlations between two phenotypes. An advantage of this method is that genetic correlations between two phenotypes can be estimated even when these phenotypes are not measured in the same individuals.

Univariate and bivariate GREML were carried out as part of sensitivity and simulation analyses. For comparison with GSEMs, GRMs were derived from directly genotyped SNPs but excluded individuals with a pairwise relationship > 0.025, as recommended (13). All analyses were conducted with GCTA software version 1.25.2 (12).

### OpenMx SEM Models

OpenMx SEM models (20), as implemented in the OpenMx software (versions 2.5 and 2.7; http://openmx.psyc.virginia.edu/), were fitted using FIML and mxGREML and included a full Cholesky decomposition of both genetic and residual variances (AE model; see above). Bivariate OpenMx SEM analyses were conducted as part of a simulation analysis. Genetic variances, genetic covariances, and genetic correlations were derived as described for GSEM above. All analyses were conducted on high-performance clusters at the University of Bristol and the Max Planck Institute for Psycholinguistics.

### Data Simulation

To evaluate the accuracy of multivariate GSEMs, we carried out data simulations (Supplemental Methods).

### Attrition Analysis

SCDC attrition scores were generated to investigate potential sources of bias. Analyses included sample-specific estimates of genetic correlations among SCDC attrition scores and between SCDC scores and subsequent sample dropout (Supplemental Methods).

## Results

### Accuracy of Multivariate GSEM

We simulated a bivariate trait (*N* = 5000) with two standardized measures (10 replicates) (Supplemental Figure S1A and Supplemental Table S3) and confirmed the accuracy of multivariate GSEMs through comparison with GCTA and OpenMx software. All methods provided accurate estimates, with respect to genetic and residual variances and covariances as well as genetic and residual factor loadings (GSEMs and OpenMx SEM models only), with comparable root mean squared error, mean absolute deviation, and little bias (bias^2^ < 10^−3^ for all methods) (Supplemental Table S3). Computationally, multivariate OpenMx SEM models were, however, more expensive (≤ 78 GB RAM FIML version 2.5; ≤ 2694 minutes mxGREML/FIML version 2.7) than multivariate GSEMs (≤ 13 GB RAM, ≤ 301 minutes) per single bivariate replicate analysis. A comparison of computing resources is shown in Supplemental Table S4. There was also little difference between estimated OpenMx versus GSEM parameters when analyzing a trivariate simulated trait with three standardized measures, as part of a benchmark test (Supplemental Figure S1B and Supplemental Table S5). Trivariate replicate analyses using OpenMx were not considered within this study owing to computational constraints.

### Univariate Analyses

**Figure 1:**
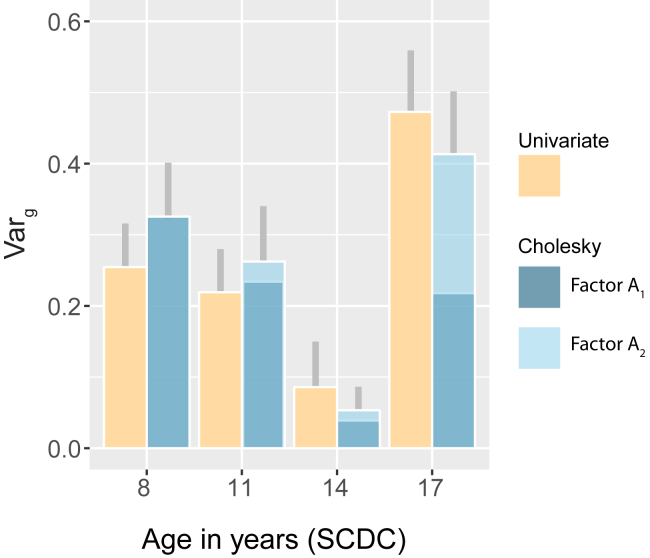
Genetic variance (Var_g_) of Social Communication Disorder Checklist (SCDC) scores during development. Var_g_ for SCDC scores across development as estimated using a univariate model (Supplemental Table S6) (*N* ≥ 4174) and the full Cholesky decomposition model (Table 1, model 1, and Supplemental Table S8) (*N* = 3295). Genetic factors A_3_ and A_4_ of the Cholesky decomposition model are not shown, as their estimated Var_g_ was negligible (< 0.01). All reported Var_g_ estimates are equivalent to SNP-h^2^ estimates. Gray lines indicate 1 SE in total Var_g_ for each SCDC measure.

Using univariate GSEMs, common genetic variants explained a large proportion of phenotypic variation in SCDC scores during childhood as well as during later adolescence (age 8, Var_g_ [SE] = 0.25 [0.061], *p* = 3.4 × 10^−5^; age 11, Var_g_ [SE] = 0.22 [0.061], *p* = 2.9 × 10^−4^; age 17, Var_g_ [SE] = 0.47 [0.086], *p* = 4.4 × 10^−8^) (Figure 1 and Supplemental Table S6) but not during early adolescence (age 14, Var_g_ [SE] = 0.086 [0.064], *p* = .18), as previously reported (2). Univariate GCTA (GREML) yielded nearly identical results (Supplemental Table S7).

### Multivariate Analyses

**Figure 2:**
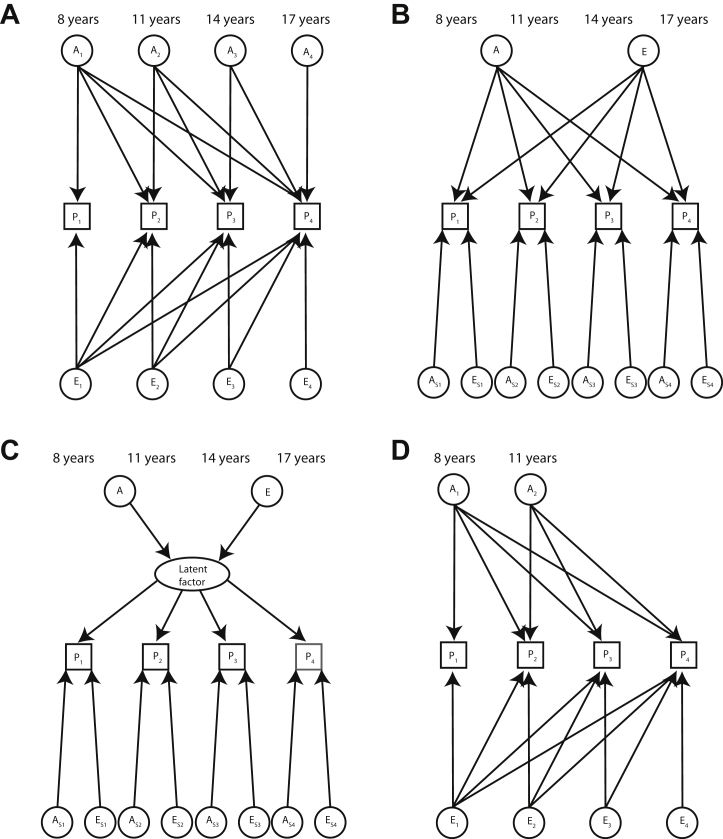
Path diagrams of a priori defined multivariate genetic-relationship-matrix structural equation models and data-driven model modifications. **(A)** Full Cholesky decomposition model. **(B)** Independent pathway model. **(C)** Common pathway model. **(D)** Two-genetic-factor Cholesky model (data-driven model modification). Observed phenotypic measures are represented by squares, and latent factors are represented by circles. Single-headed arrows (paths) define causal relationships between variables. Note that the variance of latent variables is constrained to unit variance; this is omitted from the diagrams to improve clarity.

**Figure 3:**
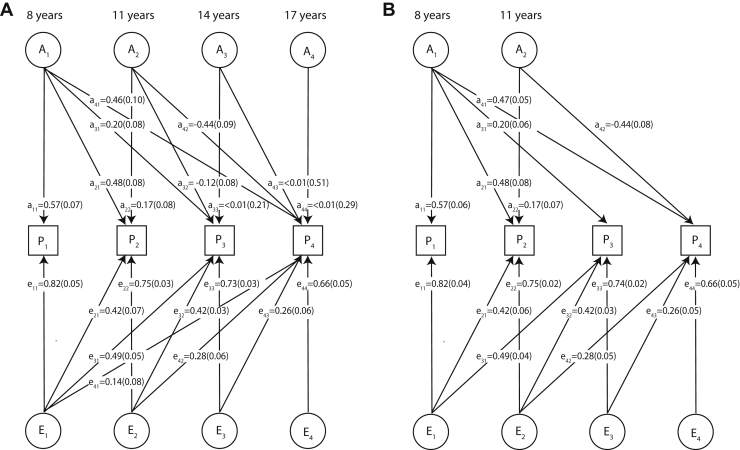
Path diagram of the full Cholesky decomposition model for Social and Communication Disorders Checklist scores **(A)** and its reduced form **(B)**. The full Cholesky decomposition model **(A)** and its most parsimonious reduced form **(B)** are described in detail in Table 1 (model 1 and 5, respectively). Corresponding to the phenotypic measures P_1_ (8 years), P_2_ (11 years), P_3_ (14 years), and P_4_ (17 years), the latent genetic factors with factor loadings (a) are A_1_ (8 years), A_2_ (11 years), A_3_ (14 years), and A_4_ (17 years), and the latent residual factors with factor loadings (e) are E_1_ (8 years), E_2_ (11 years), E_3_ (14 years), and E_4_ (17 years). All path coefficients are standardized. There were 3295 participants with repeated scores across all ages. Note that the variance of latent variables is constrained to unit variance; this is omitted from the diagrams to improve clarity.

We first examined the profile of genetic factors contributing to variation in SCDC scores during development (13,180 observations; 3295 participants) using three a priori defined multivariate GSEMs (Figure 2A–C). Based on all three fit indices, LRT, Akaike information criterion, and Bayesian information criterion, the best-fitting a priori defined model was the full Cholesky decomposition model (model 1) (Table 1, Figure 2A, and Figure 3A). Neither a single factor independent pathway model nor a single factor common pathway model could sufficiently capture the underlying variance/covariance structure of the data. As the full Cholesky decomposition model is also the baseline model, however, the model identification progressed with the identification of meaningful GSEMs through data-driven model modifications. Consistent with near zero factor loadings for the latent genetic factors A_3_ and A_4_ (Supplemental Table S8), a two-genetic-factor Cholesky model was studied (model 4) (Figure 2D) that provided a near-identical fit to the data (Δχ^2^_3_ < 0.01, *p* = 1) (Table 1). This model parameterized one genetic factor arising at 8 years of age and a second independent genetic factor explaining novel genetic influences arising at 11 years of age, each contributing to phenotypic variation during later development (Figure 2D). Using LRTs, the model fitting progressed (model 5) (Table 1 and Supplemental Table S8) until all genetic factor loadings reached *p* < 0.05 without a significant drop in the log-likelihood (Δχ^2^_2_ < 0.01, *p* = 1, with respect to model 4).

**Table 1:** Multivariate GSEMs of SCDC Scores

Model	Path Diagram	−2LL	k	Δχ^2^ to Model 1	Δ*df* to Model 1	*p*	AIC	BIC
A Priori Defined Multivariate GSEMs								
1. Full Cholesky decomposition model—saturated model	Figure 2A, Figure 3A	7900.97	20	—	—	—	7940.97	8062.97
2. Independent pathway model	Figure 2B	7914.51	16	13.55	4	.0089	7946.51	8044.12
3. Common pathway model	Figure 2C	8082.7	14	181.73	6	< 10^−15^	8110.70	8196.10
Data-Driven Model Modification								
4. Two-genetic-factor Cholesky model	Figure 2D	7900.96	17	< 0.01	3	1	7934.96	8038.67

	Path Diagram	−2LL	k	Δχ^2^ to Model 4	Δ*df* to Model 4	*p*	AIC	BIC
Best-Fitting Model								
5. Two-genetic-factor Cholesky model (excluding nonsignificant paths)^a^	Figure 3B	7900.96	15	< 0.01	2	1	7930.96	8022.47

The GSEMs were assessed with likelihood ratio tests, the AIC and the BIC. Following the investigation of a priori defined GSEM, the model fitting progressed until all genetic factor loadings reached *p* < .05 without a significant drop in the log-likelihood. Path diagrams are shown in Figure 2. There were 3295 participants with SCDC scores across all ages.

AIC, Akaike information criterion; BIC, Bayesian information criterion; GSEM, goodness-of-fit of genetic-relationship-matrix structural equation model; k, number of parameters; LL, log-likelihood; SCDC, Social and Communication Disorders Checklist.

a The best-fitting model.

The identified model included one common genetic factor A_1_ accounting for shared phenotypic variation throughout development as well as a second genetic factor A_2_ influencing SCDC scores at 11 years and especially at 17 years of age (Table 1 and Figure 3B). Figure 3 shows the full Cholesky decomposition model (model 1) and its best-fitting reduced form (model 5) with their standardized path coefficients (factor loadings ≥ 0.32 explain > 10% of the phenotypic variance).

Overall, the estimates of genetic variance, as predicted by GSEMs (model 1 and 5) (Supplemental Table S9), were consistent with univariate GSEM estimates (Figure 1), although the latter were based on larger sample numbers (Supplemental Table S6). The pattern of genetic factor loadings suggested, however, a dynamic change in the variance composition of the trait during development such that only approximately 50% of the genetic variance at 17 years of age was accounted for by genetic variation at 8 years of age (e.g., age 17, ratio Var_g_ [A_1_] to Var_g_ [A_1_+A2]; model 1, 0.53% [SE = 0.18]; model 5, 0.53% [SE = 0.12]) (Figure 1).

The predicted bivariate genetic correlations by multivariate GSEMs (model 1 and 5) (Supplemental Table S9) were overall similar to bivariate GCTA (GREML) estimates, although the latter were based on larger numbers of observations (Supplemental Table S10 and Supplemental Figure S3). Restricting analyses to the same sets of individuals, both bivariate GSEMs and bivariate GCTA (GREML) provided near-identical estimates (Supplemental Table S10), although these analyses were less powerful. Thus, small differences in genetic correlations patterns, as estimated by multivariate GSEMs versus bivariate GCTA (GREML), are likely to be due to minor differences in sample numbers.

Furthermore, there was little evidence that genetic influences between SCDC scores and subsequent SCDC sample dropout are shared in ALSPAC (Supplemental Table S11). Nominal evidence for a genetic correlation was observed between SCDC scores at 8 years of age and dropout at 14 years of age only (genetic correlation = 0.39 [SE = 0.19], *p*_*one-tailed*_ = .02). Nonetheless, SCDC attrition scores were genetically correlated across all SCDC measures in ALSPAC (*p*_*one-tailed*_ < 10^−3^) (Supplemental Table S12).

## Discussion

Using multivariate SEM in combination with common variant-based genetic correlation matrices, we investigated the developmental structure of genetic factors contributing to social communication difficulties during childhood and adolescence. We showed that the genetic architecture of this population-based complex trait changes continuously during development and is consistent with multiple genetic influences operating at different stages during development. Thus, our study provides evidence against the hypothesis that social communication behavior during development is a genetically homogeneous phenotype.

The best-fitting model, specifying two distinct genetic factors, suggested that the genetic origins of child and adolescent social communication behavior lie in middle and late childhood. The first genetic factor, parameterized to account for all genetic influences at 8 years of age, explained a considerable proportion of phenotypic variance throughout development (> 20%) with the exclusion of SCDC scores at 14 years of age that have negligible SNP-h^2^ estimates. This is consistent with recent reports of low SNP-h^2^ for autistic symptoms at the beginning of adolescence (1) and might be related to pubertal adjustments (2).

The second genetic factor, parameterized to be independent of the first one and to capture novel genetic influences arising at 11 years of age, explained predominantly phenotypic variation at 17 years of age (approximately 19%). Thus, the model predicted changes in the composition of the genetic variance during development, and only approximately 50% of the genetic variation at 17 years of age was accounted for by genetic variation at 8 years of age. Within defined developmental stages, however, such as stages spanning midchildhood to very early adolescence (e.g., 8–11 years), we found evidence for strong genetic correlations across measures. These results are consistent with recent longitudinal twin research that reported moderate to high genetic stability for autistic traits, including communication impairments, between midchildhood and early adolescence (7), but only moderate genetic stability between behavior in childhood versus emerging adulthood (8). The identified genetic factor structure using GSEMs therefore reflects both a degree of genetic stability and a genetic change in social communication behavior during development, depending on the size of the developmental window.

The identification of two distinct genetic factors, especially during later adolescence, suggests that SCDC scores at 8 or 11 years of age are, in terms of average composition, different from SCDC scores at 17 years of age. Developmental changes in the genetic architecture of social communication traits are consistent with biological maturation processes during childhood and adolescence. For example, synaptic pruning in the cerebral cortex is a signature late maturational process for generating a diversity of neuronal connections (26), which occurs during puberty and extends into early adult life (27). In parallel, there are changes in adolescent social cognitive development, especially with respect to emotional perspective taking, resistance to peer influence, and changes in social behavior (28). Given the identified genetic factor structure, it could be speculated whether multiple concepts of social reciprocity and verbal and nonverbal communication may coexist, especially at 17 years of age, and whether changes in genetic factor contributions may continue into early adulthood. Thus, even for psychological instruments with high reliability, internal consistency, and good discriminant validity, such as the SCDC (3), the nature of the captured continuous phenotype may vary across developmental periods spanning approximately 10 years. This underlines the need for behavioral genetic studies across the life span.

An important implication that flows from the observation of developmental variations in the genetic trait architecture is that measures assessed at different developmental stages may reveal different patterns of trait-disorder overlap, as previously shown for clinical autism spectrum disorder and schizophrenia (9). Moreover, the identification of a two-genetic-factor model is also consistent with recent reports of little genetic overlap between autism spectrum disorder and schizophrenia-related dimensions (29), especially with respect to social communication symptoms. Structural models capturing developmental changes in the genetic architecture of complex phenotypes can therefore be leveraged to obtain prior information concerning the stability of trait-disorder overlap and consequently the extent to which development-specific genetic trait factors are shared among different psychiatric dimensions. Our findings therefore have specific relevance for the study of functional dimensions of human behavior spanning the continuum from normal to abnormal and across development, consistent with the framework of Research Domain Criteria (30).

Finally, our study proves that structural models of genetic influences in unrelated individuals, as captured by GRMs, are computationally feasible within a longitudinal context. Beyond the scope of bivariate GCTA (GREML), multivariate GSEMs allow for the modeling of complex latent genetic factor structures across different stages of development, in particular, their genetic variance composition, and can reveal developmental origins of genetic variation that are otherwise hidden. It is furthermore possible to envisage that the concept of GSEM can be extended to investigate multivariate models of cross-disorder overlap and other complex phenomena, such as reciprocal causation. Note that also novel OpenMx FIML and mxGREML algorithms are currently being developed.

A limitation of our study is the analysis of nonmissing data across all repeatedly assessed measures. Thus, weaker genetic links, spanning wider age gaps, may not have been sufficiently captured as a consequence of lower power, although genetic correlations predicted by multivariate GSEM and bivariate GCTA(GREML) were overall similar. In addition, cohort studies can be affected by attrition bias (11). We identified, however, little evidence for a specific genetic link between variation in SCDC scores and subsequent sample dropout, although attrition scores across all assessed SCDC measures were genetically correlated. This is consistent with studies reporting an association between study nonparticipation, including SCDC dropout, and polygenic risk for schizophrenia 9, 11, regardless of when phenotypes were sampled during development. In addition, we exclusively studied rank-transformed phenotypes to ensure multivariate normality and comparability across different estimation algorithms, and we therefore cannot exclude transformation-related biases. However, genetic overlap with psychiatric conditions provided some evidence for the content validity of the analyzed trait (9). Also, maternal characteristics may have contributed to phenotypic and, to a lesser extent, genetic correlations. However, the impact of these effects is likely to be small, given the identified developmental changes in genetic variances and covariances for SCDC scores during development. Finally, a Cholesky decomposition of a variance/covariance matrix may not always result in fitting statistics that follow the expected χ^2^ distribution (31). Model comparisons using real and simulated data, however, provided little evidence for systematic differences between GCTA(GREML), GSEM, and OpenMx SEMs. Thus, despite potential limitations, our study demonstrates that structural models of longitudinally assessed behavioral traits can provide information on developmental changes in genetic trait architectures as tagged by common SNPs.

### Conclusions

The genetic architecture of social communication difficulties, as tagged by common genetic variation, changes with age and involves multiple genetic factors operating at different developmental stages during a 10-year period spanning childhood and adolescence. The identification of distinct genetic trait factors is consistent with different profiles of trait-disorder overlap and underlines the importance of investigating genetic trait variances within a multivariate context.
