# Supplementary material for: Developmental Changes Within the Genetic Architecture of Social Communication Behavior: A Multivariate Study of Genetic Variance in Unrelated Individuals
Source: Biol Psychiatry. 2018 Apr 1;83(7):598–606. doi: 10.1016/j.biopsych.2017.09.020 (PMC5855319; doi:10.1016/j.biopsych.2017.09.020)
Supplement: Supplemental Material [file mmc1.pdf]

Developmental Changes Within the Genetic Architecture of Social Communication Behavior: A Multivariate Study of Genetic Variance in Unrelated Individuals

Supplementary Information

Supplementary Methods

- Genome-wide genotype information in the Avon Longitudinal Study of Parents and Children (ALSPAC)
- Genetic-relationship-matrix structural equation models (GSEM)
- Data simulation
- Attrition within ALSPAC
- Supplementary references
- Web resources
- R gsem package installation

Supplementary Tables

- Supplementary Table S1: Descriptives of SCDC scores
- Supplementary Table S2: Phenotypic correlation of SCDC scores
- Supplementary Table S3: Bivariate simulations
- Supplementary Table S4: Computational requirements (Bivariate simulations)
- Supplementary Table S5: Trivariate simulation
- Supplementary Table S6: Univariate GSEM of SCDC scores
- Supplementary Table S7: Univariate analysis of SCDC scores: GCTA(GREML) versus GSEM
- Supplementary Table S8: Multivariate GSEM of SCDC scores: Standardised factor loadings
- Supplementary Table S9: Multivariate GSEM of SCDC scores: Estimated genetic variances and bivariate correlations
- Supplementary Table S10: Bivariate analysis of SCDC scores: GCTA(GREML) versus GSEM
- Supplementary Table S11: Genetic correlation between SCDC scores and subsequent attrition
- Supplementary Table S12: Genetic correlations between SCDC attrition scores

Supplementary Figures

- Supplementary Figure S1: Path diagrams for simulated data sets
- Supplementary Figure S2: Bivariate simulation analyses
- Supplementary Figure S3: Bivariate genetic correlations between SCDC scores during development (bivariate GREML versus multivariate GSEM)

Supplementary Methods

Genome-wide genotype information in the Avon Longitudinal Study of Parents and Children (ALSPAC)

ALSPAC children were genotyped using the Illumina HumanHap550 quad chip genotyping platforms. The ALSPAC GWAS data was generated by Sample Logistics and Genotyping Facilities at the Wellcome Trust Sanger Institute and LabCorp (Laboratory Corporation of America) using support from 23andMe. After quality control (individual call rate>0.97, SNP call rate>0.95, minor allele frequency (MAF)>0.01, Hardy-Weinberg equilibrium (HWE) $p>10^{-7}$, and removal of individuals with cryptic relatedness and non-European ancestry), 8,237 children and 477,482 directly genotyped single nucleotide polymorphisms (SNPs) were kept within the study.

Genetic-relationship-matrix structural equation models (GSEM)

Similar to genetic restricted maximum likelihood (GREML) as implemented in genome-wide complex trait analysis (GCTA) software (1), GSEM use the genetic similarity between unrelated individuals to partition the expected phenotypic variance/covariance matrix into genetic and residual components. The model assumes that genetic and residual effects are independent, and that residual effects of different individuals are independent. A normally distributed phenotype in N unrelated individuals can thus be modelled (2) as

$$\boldsymbol{P} \sim \boldsymbol{N}(\boldsymbol{0}, \boldsymbol{G}\, \boldsymbol{\sigma_g^2} + \boldsymbol{I}(\boldsymbol{1} - \boldsymbol{\sigma_g^2})) \qquad (1)$$

where P is a N x 1 vector of phenotypes, **G** is the N x N genetic correlation matrix of pairwise genome-wide genetic correlations between unrelated individuals, and **I** is a N x N identity matrix. As in the GCTA software package (1), G is the genetic relationship matrix (GRMs) constructed from common variants present on SNP chips, and σ_g^2 is an estimate of the genetic variance captured by these SNPs, while (1-σ_g^2) is an estimate of the residual variance (σ_e^2). Thus, similar to GREML (1), the total amount of phenotypic variance captured by genotyped SNPs can be estimated by fitting a univariate GSEM. GSEM uses full information maximum likelihood (FIML) and combinations of latent factor loadings and/or factor variances which can then be used to derive estimates of genetic and residual variances, covariances and correlations (see below) (3). More generally, the statistical framework of GSEM is analogous to twin analysis methodologies (4), where SEM (3) in genetically informative samples (with known average degree of genetic resemblance) are used to model the phenotypic covariance structure (5). In twin studies, genetic and environmental influences are parametrised as latent factors. The phenotypic covariance structure is often modelled by one or more additive genetic factors A (i.e. the total additive genetic effects), one or more common environmental factors C (i.e. environmental influences affecting the phenotype in

family members in an identical way) and one or more specific environmental factors E (i.e. unique exposure of family members to environmental factors). Instead of expected genetic correlations between twin pairs based on biometrical theory (4), GSEM uses genetic relationship matrices (GRMs) for genetic covariance structure modelling. Like GREML, it describes one or more additive genetic factors A and one or more residual factors E.

Within this study, we applied, univariate GSEM and multivariate GSEM, analogous to twin analysis (4). Assuming multivariate normality, and expressing the phenotype of each individual i as a deviation from the grand mean (5), the likelihood L_i for each person can be expressed as

$$\log(L_i) = -\frac{1}{2}\log|\Sigma_i| - \frac{1}{2}P_i'\Sigma_i^{-1}P_i + c = -\frac{1}{2}\log|\Sigma_i| - \frac{1}{2}tr(P_iP_i'\Sigma_i^{-1}) \qquad (2)$$

where Σ_i is the predicted variance/covariance matrix and P_i is the vector of phenotypes for the ith individual with a grand mean of 0 (P_iP_i' is the sample covariance matrix), and c is a constant term. The log likelihood (L) is then the sum of the log likelihoods for each individual.

$$\log(\mathrm{L}) = \sum\log(L_i) \qquad (3)$$

A saturated AE model can be obtained through a full decomposition of the genetic variance and residual variance into as many latent factors as there are observed measures (Cholesky decomposition). The Cholesky decomposition of the genetic variance can be described as follows (6): For a longitudinally assessed trait P with t repeat measurements, the first phenotypic measure, P_1, is influenced by a latent genetic factor (A_1), which can also explain variance in the second and all following measures (P_2,...,P_t). The second measure (P_2) is, in addition, influenced by a second latent genetic factor A_2, explaining phenotypic variance in P_2 and all following measures (P_3,...,P_t) not yet captured by A_1, and so forth. The last measure (P_t) is, beside the latent genetic factors (A_1,...,A_{t-1}), influenced by a genetic

factor A_t, which does not explain variance within any of the previous measures (P_1,...,P_{t-1}) (4). We annotate the genetic factor loadings a (path coefficients) such that the first number indicates the direction of the effect (the variable to which the arrow points) and the second the origin of the effect (4).

The expected phenotypic covariance matrix for Z-standardised traits based on the factor model is

$$\boldsymbol{\Sigma} = \boldsymbol{\lambda}\boldsymbol{\Phi}\boldsymbol{\lambda}' + \boldsymbol{\Psi}^2 \tag{4}$$

where $\boldsymbol{\lambda}$ is a lower triangular matrix of genetic factor loadings, $\boldsymbol{\Phi}$ is a diagonal matrix of latent genetic factor variances (standardised to unit variance) such that $\boldsymbol{\Phi}$ is an identity matrix $\boldsymbol{I}$, and $\boldsymbol{\Psi}^2$ a covariance matrix of residual influences (5). It is also possible to decompose the residual variance into latent residual factors, such that

$$\boldsymbol{\Sigma} = \boldsymbol{\lambda}\boldsymbol{\Phi}\boldsymbol{\lambda}' + \boldsymbol{\zeta}\boldsymbol{\Theta}\boldsymbol{\zeta}' \tag{5}$$

where $\boldsymbol{\zeta}$ is a lower triangular matrix of residual factor loadings and $\boldsymbol{\Theta}$ is a diagonal matrix of latent residual factor variances (standardised to unit variance) such that $\boldsymbol{\Theta}$ is an identity matrix $\boldsymbol{I}$. For example, for a bivariate trait consisting of measures P_1 and P_2, assuming two genetic factors (A_1 and A_2) and two genetic factors (E_1 and E_2), the expected phenotypic covariance matrix can be expressed as follows:

$$\boldsymbol{\Sigma} = \begin{bmatrix} \sigma_{p\_1}^2 & \sigma_{p\_12} \\ \sigma_{p\_12} & \sigma_{p\_2}^2 \end{bmatrix} \tag{6}$$

with the relevant matrices

$$\boldsymbol{\lambda} = \begin{bmatrix} a_{11} & 0 \\ a_{21} & a_{22} \end{bmatrix}, \boldsymbol{\Phi} = \begin{bmatrix} 1 & 0 \\ 0 & 1 \end{bmatrix}, \boldsymbol{\zeta} = \begin{bmatrix} e_{11} & 0 \\ e_{21} & e_{22} \end{bmatrix}, \boldsymbol{\Theta} = \begin{bmatrix} 1 & 0 \\ 0 & 1 \end{bmatrix} \tag{7}$$

where $\sigma_{p\_1}^2$ and $\sigma_{p\_2}^2$ represent the phenotypic variances and σ_{p_12} the phenotypic covariance.

The bivariate AE Cholesky decomposition of two standardised measures, as described above, can be visualised by means of a path diagram (Supplementary Figure S1A) and the expected phenotypic variances and covariances can be expressed as follows:

$$\sigma_{p\_1}^2 = \sigma_{g\_1}^2 + \sigma_{e\_1}^2 = a_{11}^2 + e_{11}^2 = 1 \quad (8)$$

$$\sigma_{p\_2}^2 = \sigma_{g\_2}^2 + \sigma_{e\_2}^2 = (a_{21}^2 + a_{22}^2) + (e_{21}^2 + e_{22}^2) = 1 \quad (9)$$

$$\sigma_{p\_12} = \sigma_{g\_12} + \sigma_{e\_12} = a_{11}a_{21} + e_{11}e_{21} \quad (10)$$

where $\sigma_{g\_1}^2$ and $\sigma_{g\_2}^2$ represent the genetic variances and σ_{g_12} the genetic covariance, and $\sigma_{e\_1}^2$ and $\sigma_{e\_2}^2$ the residual variances and σ_{e_12} the residual covariance. The variance of the latent factors A_1 and A_2, and E_1 and E_2 has been standardised to unit variance and is not shown.

Estimated genetic variances and covariances can subsequently be utilised to derive genetic correlations (GSEM-r_g) between two phenotypes (7), i.e. the extent to which two phenotypes share genetic factors (ranging from -1 to 1):

$$\rho_g = \frac{\sigma_{g\_12}}{\sqrt{\sigma_{g\_1}^2 \sigma_{g\_2}^2}} \quad (11)$$

where σ_{g_12} is the genetic covariance between phenotypes 1 and 2 and ρ_g the genetic correlation.

We fitted in this work an AE Cholesky decomposition model as baseline model and not, as commonly selected in twin research, a fully saturated model. A twin design, however, inherently contains genetic information based on phenotypic twin correlations in monozygotic versus dizygotic twins. A cohort sample consisting of unrelated individuals does not. Thus, fitting a fully saturated model in a general population sample of unrelated individuals will not provide information on genetic effects within that sample.

The goodness-of-fit of GSEM to empirical data was assessed using likelihood ratio test (LRT), the Akaike Information Criterion (AIC) (8) and the Bayesian Information Criterion (BIC) (9). The LRT is based on the difference in the negative log-likelihood (−2LL) of *a priori* defined models (model 2 and 3) and the saturated model (model 1), which is asymptotically chi-squared distributed with degrees of freedom equal to the difference in parameters between the models. AIC fit indices were calculated as

$$AIC = -2LL + 2k \quad (12)$$

where LL is the log-likelihood and k is the number of free model parameters in the model, with lower AIC values indexing a better model fit (8). The BIC indices take both goodness-of-fit and parsimony of the model into account, and lower BIC values indicate a better model fit (10). The index is defined as

$$BIC = -2LL + k \log (N) \quad (13)$$

where N is the number of independent observations.

GSEM were implemented within R (Rv3.2.4) via the optim function (stats library).

Data simulation

To evaluate the accuracy of multivariate GSEM, we carried out data simulations. Assuming multivariate normality, we simulated bivariate traits with two repeated measures (i.e. two genetic factors with their variances and their covariance; two residual factors with their variances and their covariance, Supplementary Figure S1A), assuming 5000 individuals and 20,000 SNPs per genetic factor, for 10 replicates. Phenotypic variances and covariances were estimated from genetic (a) and residual (e) factor loadings as expected under an AE Cholesky decomposition model (Supplementary Figure S1A). The simulated values are detailed in Supplementary Table S3. Across replicates, we calculated the mean average deviation (MAD; i.e. the absolute deviation from the mean), the root mean squared error (RMSE, i.e. the square root of the average squared difference between each replicate estimate and the true (simulated) value), and the squared bias ($Bias^2$, i.e. the squared difference between the mean of the replicate estimates and the true simulated value). As a benchmark test, we also carried out a trivariate trait simulation with three repeated measures (i.e. three genetic factors with their variances and their covariance and three residual factors with their variances and their covariance, Supplementary Figure S1B) assuming 5000 individuals and 20,000 SNPs per genetic factor, for one replicate.

Attrition within ALSPAC

To study non-participation within ALSPAC, analysis was restricted to participants who were alive at one year of age and had information on genome-wide data available (N=7,758). Dichotomic SCDC-missingness was defined as availability of mother-reported scores at 8, 11, 14 and 17 years of age. We estimated genetic correlations between these attrition scores as well as between SCDC scores and subsequent SCDC-missingness. This is possible as genetic correlations are independent of an underlying liability scale. For simplicity, we used bivariate GREML and not multivariate GSEM for the analysis, as there was little evidence for a genetic correlation between SCDC scores and subsequent attrition (see Results).

Supplementary references

1. Yang J, Lee SH, Goddard ME, Visscher PM (2011): GCTA: A Tool for Genome-wide Complex Trait Analysis. *Am. J. Hum. Genet.* 88: 76–82.
2. Golan D, Lander ES, Rosset S (2014): Measuring missing heritability: inferring the contribution of common variants. *Proc. Natl. Acad. Sci. U. S. A.* 111: E5272-5281.
3. Bollen KA (1989): *Structural Equations with Latent Variables*, 1 edition. New York: Wiley-Blackwell.
4. Neale M, Maes HHM (2004): *Methodology for genetic studies of twins and families.*, Dordrecht: Kluwer Academic Publishers.
5. Martin NG, Eaves LJ (1977): The genetical analysis of covariance structure. *Heredity* 38: 79–95.
6. Cherny SS (2005): Cholesky Decomposition. *Encycl. Stat. Behav. Sci.*John Wiley & Sons, Ltd.
7. Falconer PDS, Mackay PTFC (1995): *Introduction to Quantitative Genetics*, 4 edition. Essex, England: Longman.
8. Akaike H (1987): Factor analysis and AIC. *Psychometrika* 52: 317–332.
9. Schwarz G (1978): Estimating the Dimension of a Model. *Ann. Stat.* 6: 461–464.
10. Maindonald J, Braun WJ (2010): *Data Analysis and Graphics Using R: An Example-Based Approach*, 3 edition. Cambridge ; New York: Cambridge University Press.
11. Yang J, Benyamin B, McEvoy BP, Gordon S, Henders AK, Nyholt DR, et al. (2010): Common SNPs explain a large proportion of the heritability for human height. *Nat. Genet.* 42: 565–569.

Web resources

ALSPAC: http://www.bris.ac.uk/alspac/researchers/data---access/data---dictionary

HPC: http://www.bristol.ac.uk/earthsciences/about/facilities/hpc.html

PLINK2: http://pngu.mgh.harvard.edu/~purcell/plink/plink2.shtml

OPENMX: http://openmx.psyc.virginia.edu/

R: https://cran.r-project.org/

GCTA: http://cnsgenomics.com/software/gcta/

GSEM: https://gitlab.gwdg.de/beate.stpourcain/gsem

R gsem package installation

```
install.packages("devtools")
devtools::install_github("hadley/devtools")
install.packages("msm")
install.packages("numDeriv")
devtools::install_git('https://gitlab.gwdg.de/beate.stpourcain/gsem')
```

#Note that ssl certificate issues during the installation can arise if the ca-certificates package on the client server is out of date

Supplementary Tables

Supplementary Table S1: Descriptives of SCDC scores

Trait	Age(years)[range]	Male/Female	Mean(SD) [range]	Kurtosis	Skewness	N
**8**	7.7(0.14)[7.5;9.3]	2842/2709	2(3.71)[0;24]	9.12	2.19	5551
**11**	10.7(0.13)[10.5;13.8]	2751/2709	1(3.51)[0;24]	10.53	2.46	5460
**14**	13.9(0.15)[13. 7;16.1]	2529/2531	1(3.59)[0;24]	9.08	2.20	5060
**17**	16.8(0.36)[16.5;18.3]	2024/2150	1(3.79)[0;24]	7.09	1.89	4174

SCDC - Social and Communication Disorders Checklist; The kurtosis for the standard normal distribution is 3 and the skewness is 0

Supplementary Table S2: Phenotypic correlation of SCDC scores

**Age in years**				
	**8**	**11**	**14**	**17**
**8**	1.00	0.61	0.50	0.38
**11**	0.57	1.00	0.56	0.41
**14**	0.49	0.57	1.00	0.51
**17**	0.39	0.45	0.56	1.00

SCDC - Social and Communication Disorders Checklist
Lower triangle: Spearman's rank correlation using pairwise complete observations; Upper triangle: Pearson product moment correlation using rank-transformed scores adjusted for age, sex and the two most significant ancestry-informative principal components

Supplementary Table S3: Bivariate simulations

Label	Sim	GCTA (GREML)			GSEM (FIML)			OpenMx 2.5 (FIML)		
		Mean(RMSE)	MAD	$Bias^2$	Mean(RMSE)	MAD	$Bias^2$	Mean(RMSE)	MAD	$Bias^2$
Var_g1	0.25	0.263(0.053)	0.039	$1.8x10^{-4}$	0.263(0.053)	0.039	$1.8x10^{-4}$	0.263(0.054)	0.039	$1.8x10^{-4}$
Var_g2	0.50	0.48(0.091)	0.077	$3.6x10^{-4}$	0.481(0.091)	0.077	$3.5x10^{-4}$	0.481(0.091)	0.077	$3.5x10^{-4}$
Cov_g	0.25	0.251(0.059)	0.055	$4.6x10^{-7}$	0.251(0.059)	0.055	$3.5x10^{-7}$	0.251(0.059)	0.055	$3.0x10^{-7}$
Var_e1	0.75	0.740(0.05)	0.038	$9.0x10^{-5}$	0.74(0.05)	0.038	$9.4x10^{-5}$	0.74(0.05)	0.038	$9.2x10^{-5}$
Var_e2	0.50	0.515(0.086)	0.071	$1.8x10^{-4}$	0.513(0.086)	0.071	$1.7x10^{-4}$	0.513(0.086)	0.071	$1.7x10^{-4}$
Cov_e	0.087	0.085(0.052)	0.048	$5.5x10^{-7}$	0.087(0.052)	0.048	$7.0x10^{-7}$	0.087(0.052)	0.048	$5.7x10^{-7}$
a_{11}	0.50	-		-	0.51(0.055)	0.041	$1.1x10^{-4}$	0.51(0.055)	0.042	$1.0x10^{-4}$
a_{21}	0.50	-		-	0.49(0.096)	0.082	$9.7x10^{-5}$	0.49(0.096)	0.082	$9.5x10^{-5}$
a_{22}	0.50	-		-	0.481(0.036)	0.025	$3.8x10^{-4}$	0.481(0.036)	0.025	$3.8x10^{-4}$
e_{11}	0.87	-		-	0.86(0.029)	0.021	$3.6x10^{-5}$	0.86(0.029)	0.022	$3.6x10^{-5}$
e_{21}	0.10	-		-	0.101(0.059)	0.054	$8.3x10^{-7}$	0.101(0.059)	0.054	$6.5x10^{-7}$
e_{22}	0.70	-		-	0.705(0.054)	0.044	$2.1x10^{-5}$	0.704(0.054)	0.044	$2.0x10^{-5}$

Bivariate trait data with two repeated measures (1 and 2, N=5000 for each trait) were simulated for 10 replicates (Supplementary Figure S1A): Absolute genetic factor loadings (a) are given with respect to two simulated genetic factors A_1 and A_2; Absolute residual factor loadings (e) are given with respect to two simulated residual factors E_1 and E_2; Simulated data were analysed with bivariate GCTA(GREML), bivariate GSEM(FIML) (full Cholesky factorisation) and bivariate OpenMx SEM (full Cholesky factorisation using the fastest OpenMx SEM algorithm, OpenMx(FIML) v2.5; see Supplementary Table S4).

$Bias^2$ - Squared bias defined as the squared difference between the mean of the replicate estimates and the true simulated value; Cov_e - Residual covariance; Cov_g - Genetic covariance; FIML - Full information maximum likelihood; GCTA - Genome-wide complex trait analysis; GREML - Genetic-relationship matrix restricted maximum likelihood; GSEM - Genetic-relationship-matrix structural equation models; MAD - Mean absolute deviation; Mean - Mean of 10 replicate estimates; RMSE - Root mean squared error; SEM - Structural equation models; Sim - Simulated (true) value; Var_e - Residual variance; Var_g - Genetic variance

Supplementary Table S4: Computational requirements (Bivariate simulations)

N	GCTA (GREML)		GSEM (FIML)		OpenMx 2.5 (FIML)[a]		OpenMx 2.7 (FIML)[a]		OpenMx 2.7 (mxGREML)[a]	
	RAM(GB)	Time(min)	RAM(GB)	Time(min)	RAM(GB)	Time(min)	RAM(GB)	Time(min)	RAM(GB)	Time(min)
**1000**	<0.1	<1	0.5	9	3.3	9	1.0	29	0.7	32
**2000**	1.0	2	1.5	98	12.8	44	3.7	213	2.4	298
**3000**	2.1	9	3.2	121	28.4	115	8.3	600	5.2	785
**4000**	4.0	25	5.6	298	50.2	204	16.0	1260	9.6	1681
**5000**	5.9	28	8.7	301	78.4	351	22.8	2694	15.1	2597

Bivariate trait data with two repeated measures were simulated for one replicate using a series of different sample sizes (1000 to 5000). The estimated parameter values are identical to those described in Supplementary Table S3. Analyses were conducted using one core (2.60 GHz) with access to 64 GB or, if required, 256 GB; RAM(GB) - Memory in Giga Bytes; Time(min) - Time in minutes; a - All OpenMx (mx) analyses were carried out with the options: options(mxCondenseMatrixSlots=TRUE) and mxOption(NULL,"Default optimizer","NPSOL"); GCTA - Genome-wide complex trait analysis; GREML - Genetic-relationship matrix restricted maximum likelihood, FIML - Full information maximum likelihood

Supplementary Table S5: Trivariate simulation

Label	Sim	GSEM (FIML) Estimate(SE)	OpenMx 2.5 (FIML) Estimate(SE)
a_{11}	0.80	0.83(0.043)	0.83(0.043)
a_{21}	0.50	0.498(0.055)	0.498(0.055)
a_{31}	0.00	-0.010(0.062)	-0.011(0.062)
a_{22}	0.40	0.403(0.04)	0.403(0.04)
a_{32}	0.50	0.465(0.071)	0.464(0.071)
a_{33}	0.10	0.101(0.165)	0.098(0.17)
e_{11}	0.60	0.572(0.058)	0.572(0.058)
e_{21}	0.60	0.625(0.06)	0.625(0.06)
e_{31}	0.20	0.211(0.086)	0.212(0.086)
e_{22}	0.48	0.458(0.04)	0.458(0.04)
e_{32}	0.50	0.537(0.067)	0.538(0.067)
e_{33}	0.67	0.682(0.026)	0.682(0.026)

A trivariate trait with three standardised measures (1, 2 and 3) was simulated (a single replicate) as a benchmark test (Supplementary Figure S1B): Absolute genetic factor loadings (a) are given with respect to three simulated genetic factors A_1, A_2 and A_3; Absolute residual factor loadings (e) are given with respect to three simulated residual factors E_1, E_2 and E_3; Simulated data were analysed with trivariate GSEM(FIML) (full Cholesky factorisation) and trivariate OpenMx SEM (full Cholesky factorisation using the fastest OpenMx SEM algorithm, OpenMx(FIML) v2.5; see Supplementary Table S4).

SEM - Structural equation models; Sim - Simulated (true) value; GSEM - Genetic-relationship-matrix structural equation models

Supplementary Table S6: Univariate GSEM of SCDC scores

	GSEM		
**Age(y)**	**Var_g(SE)**	***p***	**N**
8	0.25(0.06)	3.36×10^{-5}	5551
11	0.22(0.06)	2.94×10^{-4}	5460
14	0.086(0.06)	0.18	5060
17	0.47(0.09)	4.40×10^{-8}	4174

GSEM - Genetic-relationship-matrix structural equation models; Var_g - Additive genetic variance; SCDC - Social and Communication Disorders Checklist

Univariate GSEM was carried out by estimating the genetic variance as a variance component without constraints (2-sided test). The reported Var_g estimates are equivalent to SNP-h^2 estimates due to the standardisation of the analysed traits.

Supplementary Table S7: Univariate analysis of SCDC scores: GCTA(GREML) versus GSEM

	**GCTA(GREML)**			**GSEM**		
**Age**	**Var_g(SE)**	***p***	**N**	**Var_g(SE)**	***p***	**N**
8	0.23(0.07)	$1.6x10^{-4}$	4971	0.23(0.07)	$1.6x10^{-4}$	4971
11	0.15(0.07)	0.011	4895	0.15(0.07)	0.011	4895
14	0.097(0.07)	0.077	4566	0.099(0.07)	0.077	4566
17	0.47(0.09)	$5.0x10^{-9}$	3779	0.47(0.09)	$5.0x10^{-9}$	3779

Age - Age in years; GCTA - Genome-wide complex trait analysis; GREML - Genetic-relationship matrix restricted maximum likelihood; GSEM - Genetic-relationship-matrix structural equation models; SCDC - Social and Communication Disorders Checklist; Var_g - Additive genetic variance

Differences compared with the total sample N are due to the exclusion of individuals with a relatedness of ≥ 2.5% from all ALSPAC participants with genome-wide data, allowing for identical sample numbers across GREML and GSEM analyses. The reported Var_g estimates are equivalent to SNP-h^2 estimates due to the standardisation of the analysed traits.

Univariate GSEM was carried out by estimating the genetic variance without constraints. Note, that the GREML likelihood ratio test, as implemented in GCTA (11), follows by default a 50:50 mixture distribution with a point mass at 0 and a chi-squared distribution (df=1), which is comparable to a one-tailed *p*-value. For comparison with GSEM, however, an unconstrained GCTA option was selected.

Supplementary Table S8: Multivariate GSEM of SCDC scores: Standardised factor loadings

	Full Cholesky decomposition (Model 1)		Best-fitting model (Model 5)	
**Label**	**Estimate(SE)**	***p***	**Estimate (SE)**	***p***
**a_{11}**	0.57(0.07)	$5.09x10^{-13}$	0.57(0.06)	$8.69x10^{-17}$
**a_{21}**	0.48(0.08)	$1.30x10^{-8}$	0.48(0.08)	$2.57x10^{-9}$
**a_{31}**	0.20(0.08)	$2.15x10^{-2}$	0.20(0.06)	$4.10x10^{-3}$
**a_{41}**	0.46(0.10)	$2.15x10^{-6}$	0.47(0.05)	$3.29x10^{-21}$
**a_{22}**	0.17(0.08)	$4.09x10^{-2}$	0.17(0.07)	$1.94x10^{-2}$
**a_{32}**	-0.12(0.08)	$1.69x10^{-1}$	-	-
**a_{42}**	-0.44(0.09)	$2.60x10^{-6}$	-0.44(0.08)	$8.24x10^{-8}$
**a_{33}**	<0.01(0.21)	1	-	-
**a_{43}**	<0.01 (0.51)	1	-	-
**a_{44}**	<0.01 (0.29)	1	-	-
**e_{11}**	0.82(0.05)	$<10^{-10}$	0.82(0.04)	$<10^{-10}$
**e_{21}**	0.42(0.07)	$1.73x10^{-9}$	0.42(0.06)	$<10^{-10}$
**e_{31}**	0.49(0.05)	$<10^{-10}$	0.49(0.04)	$<10^{-10}$
**e_{41}**	0.14(0.08)	$6.69x10^{-2}$	-	-
**e_{22}**	0.75(0.03)	$<10^{-10}$	0.75(0.02)	$<10^{-10}$
**e_{32}**	0.42(0.03)	$<10^{-10}$	0.42(0.03)	$<10^{-10}$
**e_{42}**	0.28(0.06)	$1.13x10^{-6}$	0.28(0.05)	$1.55x10^{-7}$
**e_{33}**	0.73(0.03)	$<10^{-10}$	0.74(0.02)	$<10^{-10}$
**e_{43}**	0.26(0.06)	$1.51x10^{-5}$	0.26(0.05)	$8.28x<10^{-9}$
**e_{44}**	0.66(0.05)	$<10^{-10}$	0.66(0.05)	$<10^{-10}$

The full Cholesky decomposition model and its best-fitting reduced form are described in Table 1 (Model 1 and model 5 respectively) and Figure 3: Genetic factor loadings *a* are given with respect to the latent genetic factor A_1(8 years), factor A_2(11 years), factor A_3(14 years) and factor A_4(17 years) and residual factor loadings *e* with respect to factor E_1(8 years), factor E_2(11 years), factor E_3(14 years) and factor E_4(17 years). 3,295 participants had non-missing scores across all ages.

GSEM - Genetic-relationship-matrix structural equation models; SCDC - Social and Communication Disorders Checklist

Supplementary Table S9: Multivariate GSEM of SCDC scores: Estimated genetic variances and bivariate correlations

**Full Cholesky decomposition (Model 1)**				
**Var_g(SE)**				
**Age**	**8**	**11**	**14**	**17**
	0.32(0.08)	0.26(0.08)	0.05(0.03)	0.41(0.09)
***r_g*(SE)**				
**Age**	**8**	**11**	**14**	**17**
**8**	1	-	-	-
**11**	0.95(0.05)	1	-	-
**14**	0.86(0.20)	0.65(0.29)	1	-
**17**	0.73(0.12)	0.46(0.16)	0.98(0.08)	1
**Best-fitting model (Model 5)**				
**Var_g(SE)**				
**Age**	**8**	**11**	**14**	**17**
	0.32(0.07)	0.26(0.07)	0.04(0.03)	0.41(0.07)
***r_g*(SE)**				
**Age**	**8**	**11**	**14**	**17**
**8**	1	-	-	-
**11**	0.95(0.05)	1	-	-
**14**	1.00(<0.01)	0.95(0.05)	1	-
**17**	0.73(0.08)	0.46(0.13)	0.73(0.08)	1

The full Cholesky decomposition model and its best-fitting reduced form are described in Table 1 (Model 1 and model 5 respectively) and Figure 3. 3,295 participants had non-missing scores across all ages.

Age - Age at measurement in years; GSEM - Genetic-relationship-matrix structural equation models; r_g - Genetic correlation; SCDC - Social and Communication Disorders Checklist; Var_g - Genetic variance

Note that SCDC scores at 14 years were retained within the model, irrespective of their low SNP-heritability, due to their genetic correlations with other SCDC measures earlier and later during development. The reported Var_g estimates are equivalent to SNP-h^2 estimates and based on standardised path coefficients.

Supplementary Table S10: Bivariate analysis of SCDC scores: GCTA(GREML) versus GSEM

**Full**	**Age**	**8 vs 11 Obs=9,866**	**8 vs 14 Obs =9,537**	**8 vs 17 Obs =8,750**	**11 vs 14 Obs =9,461**	**11 vs 17 Obs =8,674**	**14 vs 17 Obs =8,345**
**Method**		**Estimate(SE)**	**Estimate(SE)**	**Estimate(SE)**	**Estimate(SE)**	**Estimate(SE)**	**Estimate(SE)**
**GCTA(GREML)**	A_1	0.22(0.065)	0.22(0.067)	0.23(0.068)	0.17(0.067)	0.16(0.068)	0.09(0.07)
	A_2	0.15(0.065)	0.09(0.069)	0.47(0.087)	0.11(0.069)	0.48(0.086)	0.49(0.086)
	Cov_g	0.17(0.053)	0.1(0.052)	0.15(0.056)	0.11(0.055)	0.11(0.057)	0.17(0.061)
	r_g	0.95(0.156)	0.68(0.266)	0.46(0.145)	0.79(0.217)	0.40(0.17)	0.82(0.257)
	$\boldsymbol{p}_{one\text{-}tailed}$	0.00030	0.025	0.0025	0.018	0.024	0.00014
**Non-missing**	**Age**	**8 vs 11 Obs =8,434**	**8 vs 14 Obs =7,872**	**8 vs 17 Obs =6,696**	**11 vs 14 Obs =8,820**	**11 vs 17 Obs =6,942**	**14 vs 17 Obs =7,014**
**Method**		**Estimate(SE)**	**Estimate(SE)**	**Estimate(SE)**	**Estimate(SE)**	**Estimate(SE)**	**Estimate(SE)**
**GCTA(GREML)**	A_1	0.16(0.075)	0.3(0.082)	0.22(0.092)	0.13(0.076)	0.11(0.088)	0.03(0.089)
	A_2	0.08(0.077)	0.07(0.081)	0.41(0.099)	0.01(0.076)	0.47(0.096)	0.44(0.091)
	Cov_g	0.11(0.062)	0.12(0.064)	0.15(0.072)	0.04(0.062)	0.08(0.07)	0.11(0.071)
	r_g	1(0.354)	0.89(0.418)	0.51(0.188)	-	0.33(0.243)	-
	$\boldsymbol{p}_{one\text{-}tailed}$	0.5	0.019	0.013	-	0.13	-
**GSEM**	A_1	0.17(0.075)	0.3(0.081)	0.22(0.091)	0.13(0.074)	0.11(0.084)	0.04(0.037)
	A_2	0.1(0.061)	0.07(0.078)	0.41(0.094)	0.02(0.054)	0.47(0.092)	0.47(0.092)
	Cov_g	0.13(0.06)	0.13(0.062)	0.15(0.069)	0.05(0.058)	0.08(0.067)	0.14(0.065)
	r_g	1(0)	0.89(0.422)	0.51(0.182)	-	0.33(0.229)	-

Age - Age at measurement in years; GCTA - Genome-wide complex trait analysis; GREML - Genetic-relationship matrix restricted maximum likelihood; GSEM - Genetic-relationship-matrix structural equation models; SCDC - Social and Communication Disorders Checklist

Bivariate analyses were conducted using bivariate GCTA(GREML) and bivariate GSEM (full Cholesky decomposition of the genetic and residual variance). Analyses were either carried out using the full phenotypic information (GCTA(GREML) only; including individuals with information at one time point only), or restricting the analysis to individuals with non-missing information across both time points. Individuals with a relatedness of $\geq$ 2.5% were excluded. For comparisons, genetic variances (Var_g), covariances (Cov_g) and correlations (r_g) are shown. r_g estimates for traits with Var_g<0.05 are not reported due to large estimation errors.

Supplementary Table S11: Genetic correlation between SCDC scores and subsequent attrition

**r_g(SE), $p_{one\text{-}tailed}$**	**8 (Score)**	**11 (Score)**	**14 (Score)**	**17 (Score)**
**8 (miss)**	-	-	-	-
**11 (miss)**	0.33(0.23), *p*=0.06	-	-	-
**14 (miss)**	0.39(0.19), *p*=0.02	0.27(0.27), *p*=0.15	-	-
**17 (miss)**	0.24(0.20), *p*=0.11	0.20(0.28), *p*=0.24	0.28(0.34), *p*=0.19	-

miss - Missingness in Social and Communication Disorders Checklist (SCDC) scores; Scores - SCDC scores

Genetic correlations (r_g) were estimated using bivariate genetic-relationship matrix restricted maximum likelihood (GREML) as implemented in genome-wide complex trait analysis (GCTA) software

Supplementary Table S12: Genetic correlations between SCDC attrition scores

r_g(SE), $p_{one\text{-}tailed}$ Miss	8	11	14	17
**8**	-	-	-	-
**11**	0.81(0.13), p=4.8x10^{-4}	-	-	-
**14**	0.83(0.12) , p=2.2x10^{-5}	0.84(0.11), p=1.1x10^{-4}	-	-
**17**	1.00(0.15) , p=8.4x10^{-7}	1.00(0.15), p=2.2x10^{-5}	0.91(0.09), p=3.3x10^{-6}	-

Miss - Missingness in Social and Communication Disorders Checklist (SCDC) scores

Genetic correlations (r_g) were estimated using bivariate genetic-relationship matrix restricted maximum likelihood (GREML) as implemented in genome-wide complex trait analysis (GCTA) software

Supplemental Figures

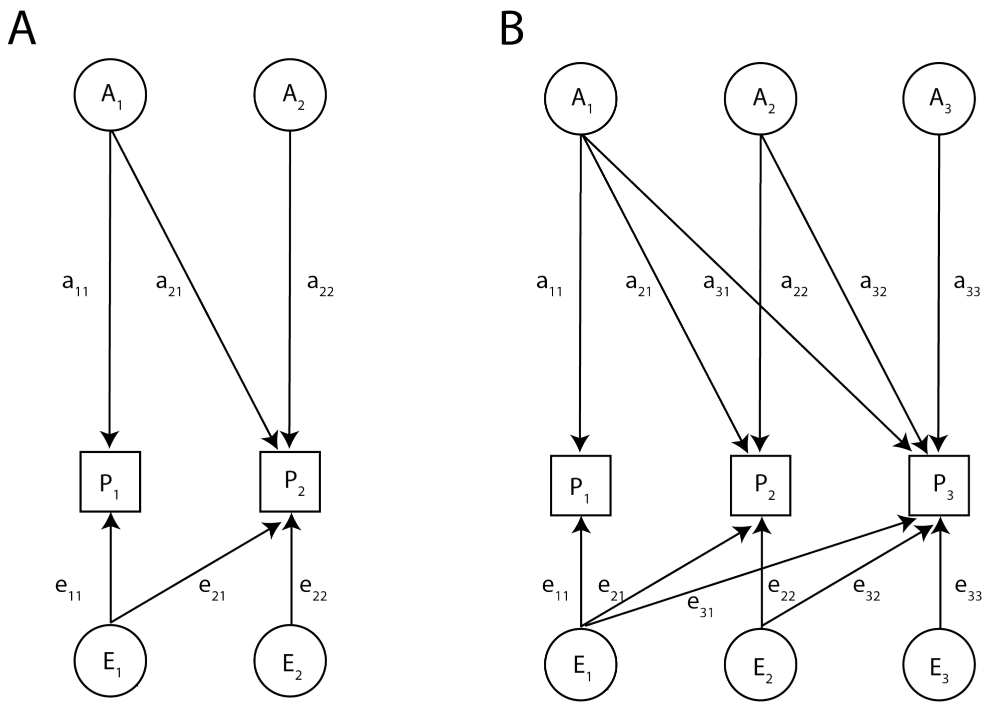

Supplementary Figure S1: Path diagrams for **s**imulated data sets

A - A bivariate trait consisting of two standardised measures P_1 and P_2 was simulated for two genetic factors (A_1 and A_2) and two residual factors (E_1 and E_2), shown with genetic and residual factor loadings, assuming 5000 individuals and 20,000 SNPs per genetic factor (10 replicates)

B - A trivariate trait consisting of three standardised measures P_1, P_2 and P_3 was simulated for three genetic factors (A_1, A_2 and A_3) and three residual factors (E_1, E_2 and E_3), shown with genetic and residual factor loadings, assuming 5000 individuals and 20,000 SNPs per genetic factor (one replicate)

Observed phenotypic measures are represented by squares, while latent factors are represented by a circle. Single headed arrows ('paths') denote causal relationships between variables and are shown for genetic factor loadings (a) and residual factor loadings (e). Note that the variance of latent variables is constrained to unit variance, this is omitted from the diagrams to improve clarity.

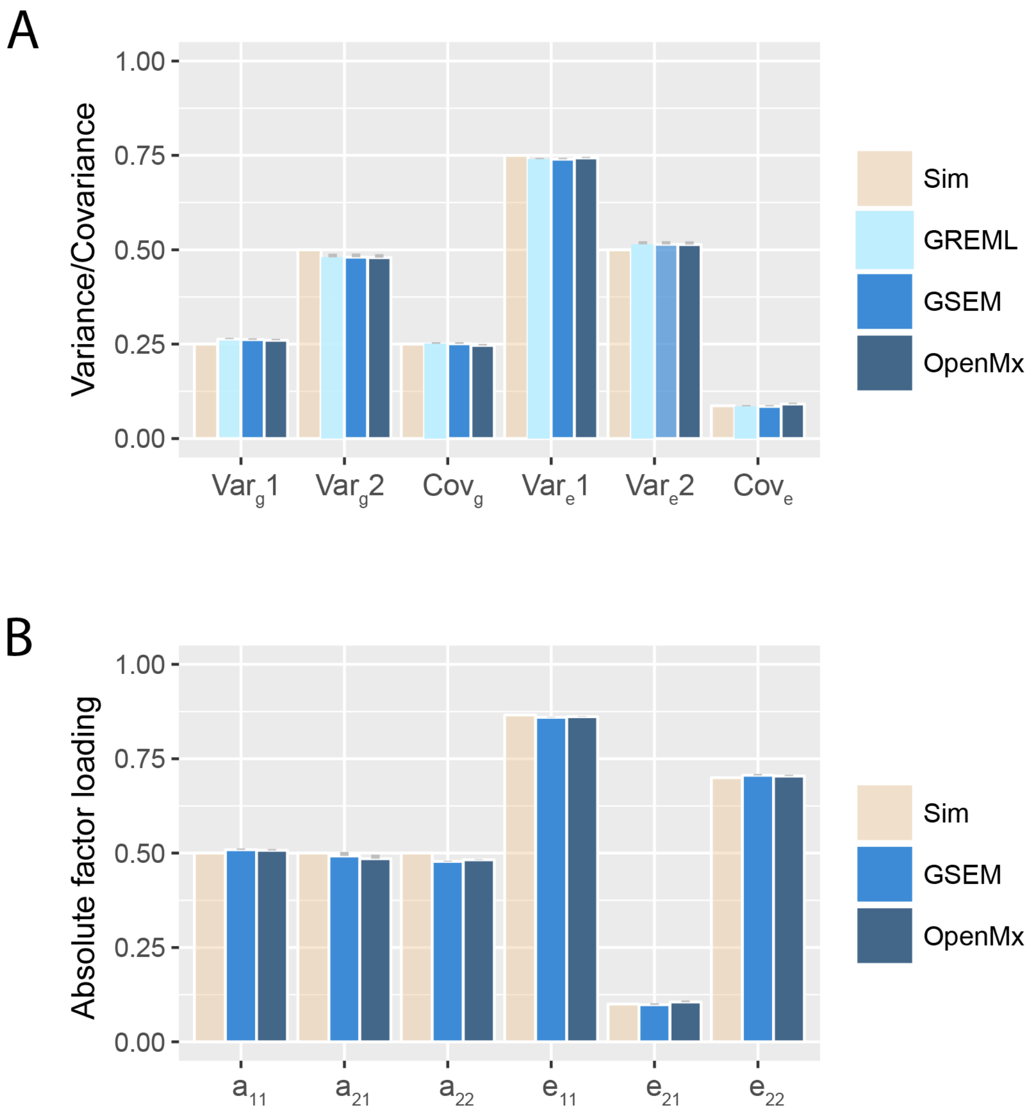

Supplementary Figure S2: Bivariate simulation analyses

Mean estimated genetic and environmental variances and covariances (A) and factor loadings (B) are shown for 10 replicates with mean squared errors as grey bars. Simulated data were analysed with GCTA(GREML), GSEM (full Cholesky factorisation) and OpenMx SEM (full Cholesky factorisation, OpenMx 2.5, FIML). Bivariate traits with two standardised measures (1 and 2) were simulated assuming two genetic factors (A_1 and A_2) and two residual factors (E_1 and E_2; Supplementary Figure S1A, Supplementary Table S3).

a - Genetic factor loading; Cov_e - Residual covariance; Cov_g - Genetic covariance; e - Residual factor loading; FIML - Full information maximum likelihood; GCTA - Genome-wide complex trait analysis; GREML - Genetic-relationship-matrix residual maximum likelihood; GSEM - Genetic-relationship-matrix structural equation models; SEM - Structural equation models; Sim - Simulated value; Var_e - Residual variance; Var_g - Genetic variance

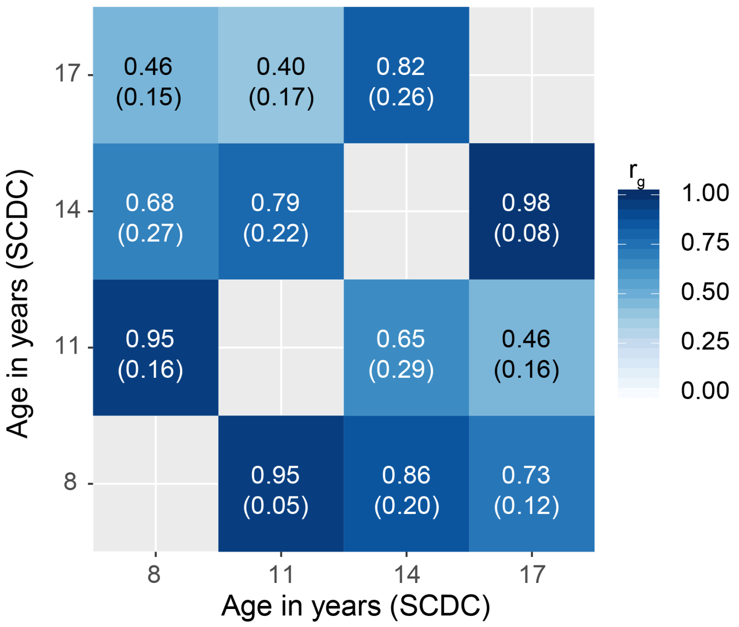

Supplementary Figure S3: Bivariate genetic correlations between SCDC scores during development (bivariate GREML versus multivariate GSEM)

Genetic correlations (r_g) were estimated with multivariate GSEM (lower triangle; as shown for the full Cholesky decomposition model in Supplementary Table S9, N=13,180 observations) and bivariate GCTA(GREML) (upper triangle; Supplementary Table S10, N≤ 9,866 observations; analyses using full phenotypic information) and are shown with their standard errors.

Note that both bivariate GREML and bivariate GSEM analyses using identical sample numbers provided nearly identical r_g estimates (Supplementary Table S10, non-missing sample analyses).

GCTA - Genome-wide complex trait analysis; GREML - Genetic-relationship matrix restricted maximum likelihood; GSEM - Genetic-relationship-matrix structural equation models
